# Treatment of Stage 2 Medication-Induced Osteonecrosis of the Jaw: A Case Series

**DOI:** 10.3390/ijerph18031018

**Published:** 2021-01-24

**Authors:** Guillermo Pardo-Zamora, Yanet Martínez, Jose Antonio Moreno, Antonio J. Ortiz-Ruíz

**Affiliations:** Department of General Dentistry and Implants, Faculty of Medicine and Dentistry, University of Murcia, 30008 Murcia, Spain; yanet.martinez@um.es (Y.M.); joseantonio171087@gmail.com (J.A.M.); ajortiz@um.es (A.J.O.-R.)

**Keywords:** osteonecrosis, medication-induced maxillary osteonecrosis, bisphosphonates, denosumab, osteonecrosis treatment

## Abstract

Medication-induced jaw osteonecrosis (MRONJ) is a rare and serious disease with a negative impact on patients’ quality of life, whose exact cause remains unclear and which may have a multifactorial origin. Although there are different therapeutic protocols, there is still no consensus. This case series evaluated three patients diagnosed with staged 2 MRONJ treated at the University of Murcia dental clinic according to the protocols described by the Spanish Society of Oral and Maxillofacial Surgery and the American Association of Oral and Maxillofacial Surgeons. Within 12 months of the application of therapeutic protocols, the lesions were completely healed in all cases. Radiography showed slow but progressive healing with normal bone structure. Conservative treatment with antibiotics, chlorhexidine rinses and minimally invasive surgical intervention with necrotic bone resection is effective in treating stage 2 of MRONJ. In cases of refractory osteonecrosis, the application of platelet and leukocyte-rich fibrin (PRF-L) in the surgical approach improves the outcome in soft tissue healing and bone regeneration but further research is needed to confirm its effectiveness.

## 1. Introduction

Medication-related osteonecrosis of the jaw (MRONJ) is an uncommon but serious debilitating disease, whose cause remains unclear, although it may have a multifactorial origin [1]. MRONJ is characterized by exposed bone that does not heal in patients with a history or continued use of anti-resorptive or antiangiogenic agents and without a history of exposure to radiation in the head and neck [2,3].

The first cases of MRONJ were described by Marx during the 2000s in patients receiving bisphosphonates (BPS) [4], and was named bisphosphonate-related osteonecrosis of the jaw (BRONJ) [5]. However, from 2010 onwards, an increase in the prevalence of osteonecrosis was observed in patients treated with anti-resorptive and antiangiogenic drugs other than bisphosphonates, such as denosumab, and this was named denosumab-related osteonecrosis of the jaw (DRONJ) [6,7]. In 2014, the American Association of Oral and Maxillofacial Surgeons (AAOMS) recommended replacing the name BRONJ with MRONJ [8].

Anti-resorptive drugs such as BPS modulate bone metabolism by inhibiting bone reabsorption, and limiting the activity of osteoclasts [9], although they also have an antiangiogenic effect [10]. They are effective in the treatment of diseases such as osteoporosis, bone metastases, multiple myeloma and Paget’s disease, among others [11,12,13]. In addition to BPS, other anti-resorptive drugs are used to treat these diseases, including denosumab, a RANKL inhibitor that is a monoclonal antibody which inhibits osteoclast function and, therefore, bone reabsorption. It is administered subcutaneously every 6 months to decrease the risk of fractures in patients with osteoporosis [14] and monthly in bone metastases of solid tumors [8]. Unlike BPS, RANKL inhibitors do not bond with the bone and their effects on bone remodeling are mostly lessened 6 months after treatment discontinuation [8,15].

Antiangiogenic drugs interfere with the formation of new blood vessels by binding to various signaling molecules and interrupting the angiogenesis signaling cascade. These new drugs have proven effective in treating gastrointestinal tumors, renal cell carcinomas, neuroendocrine tumors and other malignancies, but favor the onset of MRONJ [8].

According to the AAOMS, a patient is considered to have MRONJ if all the following conditions are met:

- Current or prior treatment with anti-resorptive or antiangiogenic agents.
- Maxillofacial intra- or extraoral bone fistula persisting for >8 weeks.
- No history of maxillary radiation therapy or bone metastases [8].

The AAOMS also defines the following grades or stages (Table 1):

Various treatments are recommended for the different grades or stages of MRONJ (Table 2) [8]:

Regardless of the disease stage, mobile bone fragments should be removed without exposing unaffected bone. The extraction of symptomatic teeth within the exposed necrotic bone should be considered because extraction is unlikely to exacerbate the necrotic process [8].

The Spanish Society of Oral and Maxillofacial Surgery (SECOM) recommends the following protocol for the treatment of MRONJ (Table 3) [16]:

Risk factors for MRONJ include not only medication and dental interventions, but also demographic, systemic and genetic factors [17]. The highest prevalence is in females in the peri- or postmenopausal period or where there is a decrease in estrogen levels, leading to alterations in bone homeostasis, although this may also be a reflection of the underlying disease for which pharmacological agents (osteoporosis, breast cancer) have been prescribed. Patients with cancer, anemia and diabetes have a higher risk of MRONJ [18]. 

Genetically, studies have reported that single nucleotide polymorphisms are associated with an increased risk of MRONJ. A 2012 gene study showed that patients with a single nucleotide polymorphism in the RBMS3 gene, which is associated with bone density and collagen formation, had a 5.8-fold risk of jaw osteonecrosis [19]. Another study analyzed polymorphisms related to the activity of farnesyl diphosphate synthase, an enzyme specifically inhibited by bisphosphonates, and found a positive correlation between carrier status and jaw osteonecrosis [8,20].

Smoking has been recognized in the literature as a risk factor for MRONJ. In the oral cavity, carcinogens in smoking delay wound healing and worsen periodontal disease, as well as promote soft tissue epithelial changes and cancer. In addition to affecting healing, nicotine in smoking may cause increased vasoconstriction in bone, leading to ischemic states that may underlie the pathophysiology of osteonecrosis [21]. However, there is no sufficient scientific evidence to corroborate it. For example, in a case-control study, smoking was assessed as a risk factor for MRONJ in cancer patients, but was not statistically significant [22]. In another more recent case-control study, no direct association of smoking with MRONJ was found in a sample of cancer patients exposed to zolendronate [18]. Vahtsevanos et al. did not observe an association between smoking and MRONJ [23]. More studies with statistically significant results are needed to determine whether tobacco should be considered a risk factor or not.

Studies comparing the effects of denosumab and bisphosphonates found a similar incidence of MRONJ [24]. Denosumab (DMAb) is a monoclonal antibody directed against RANKL and exerts its activity by forming a complex with RANKL and thus inhibiting the activation of its descending pathway [25]. Based on this premise, it is possible to consider that the mechanism of action of denosumab mimics the activity of osteoprotegerin (OPG), a soluble anti-RANKL decoy receptor that is physiologically produced by osteoblasts and inhibits osteoclasts. Therefore, an important difference compared to bisphosphonates is represented by the fact that denosumab is specific for the RANKL pathway. Furthermore, due to the different mechanisms of action, it is assumed to have a time-limited effect. On the other hand, bisphosphonates (BP) have a chemical similarity with pyrophosphate sharing the presence of two phosphate groups within the molecule. BPs can be divided into two classes: BP that do not contain nitrogen and that contain nitrogen [26].

Non-nitrogen containing BPs are represented by clodronate and etidronate. These compounds were discovered earlier, but they are less active and for this reason their use is substantially restricted to bone metabolic disorders. They exert an antiresorbent effect that is obtained by competing with the recruitment of intracellular ATP in osteoclasts, thus blocking the energy reserve of these cells, inducing their apoptosis [27].

Nitrogen-containing BPs such as zoledronate (ZA) and pamidronate were developed later, exhibit stronger activity and are predominantly used in neoplastic bone diseases, but lower doses are also prescribed in benign conditions. The mechanisms of action of these drugs have not been fully determined. However, those that present nitrogen has been shown to be more potent and bind and inhibit key enzymes of the intracellular mevalonate pathway, thus preventing the prenylation and activation of small GTPases that are essential for bone resorption activity and survival. osteoclasts [28]. The final effect is similar to that observed with non-nitrogenous family members and is represented by apoptosis of osteoclasts [29] Denosumab and bisphosphonates have differing pharmacokinetics, resulting in the treatment of osteonecrosis caused by denosumab being more effective. The main factor is that the half-life of bisphosphonates is approximately 10 years, while denosumab is eliminated from the body 6 months after treatment cessation, allowing a faster resolution of osteonecrosis and decreasing the risk after drug discontinuation [24]. We believe that more studies are necessary to assess in short periods of time (less than 3 months) whether the effects of denosumab are comparable to those of BP. In longer periods of 6 months or more, it does seem to be associated with an increased risk of MRONJ compared to bisphosphonates. However, it has been seen that cessation of denosumab therapy may be associated with a faster resolution rate of MRONJ than that of BP; although this requires more prospective studies [30].

In a systematic analysis conducted with a total of 8963 patients, it was determined that the use of denosumab was associated with a significantly higher risk of MRONJ compared to treatment with bisphosphonates (BP) [31].

On the other hand, Limones et al. In a systematic review and meta-analysis published in 2020, they conclude that the use of denosumab is associated with a higher risk of developing maxillary osteonecrosis when compared to zolendronic acid (bisphosphonate), obtaining statistically significant differences between denosumab and BP to develop MRONJ after 1 year (*p* = 0.030), 2 years (*p* = 0.006) and 3 years of exposure (*p* = 0.007) [32].

The objective of this study was to describe the results obtained in three patients diagnosed with MRONJ treated by the University of Murcia dental clinic according to the SECOM [16] and AAOMS [8] protocols.

## 2. Case Reports

### 2.1. Case 1: Conservative Treatment

**Figure 1:**
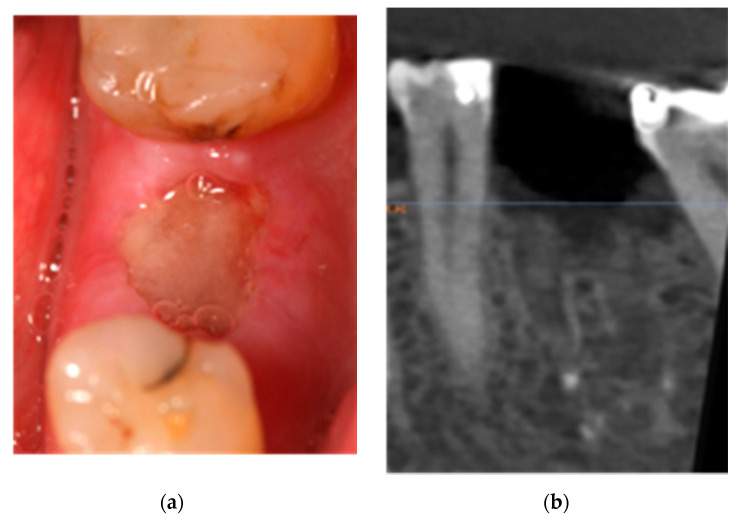
(**a**) Clinical aspect of the injury. (**b**) X-ray of the affected area.

A 60-year-old woman smoker diagnosed with breast cancer, had received oral alendronate (Fosamax weekly, MSD, Madrid, Spain) for 10 months to combat osteoporosis caused by ovarian suppression, chemotherapy treatment, and the postmenopausal state itself. She presented discomfort and pain in the left hemimandible 10 weeks after removal of the first left lower molar. The examination showed an area with exposed necrotic bone measuring 8 × 6 mm^2^ with exudate (Figure 1); the patient was diagnosed with stage 2 MRONJ.

**Figure 2:**
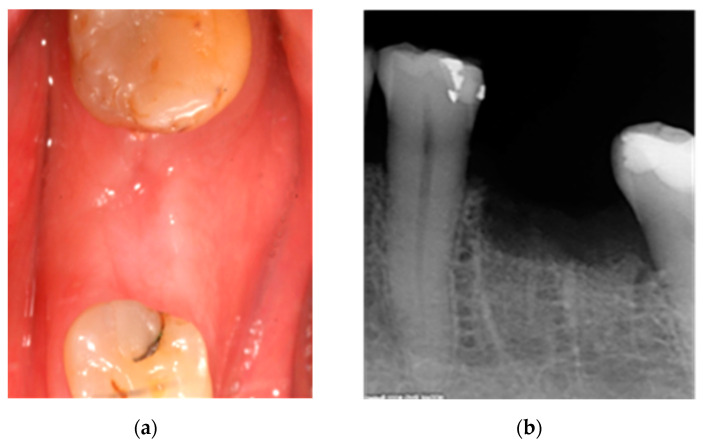
(**a**) Clinical aspect and (**b**) X-ray of the affected area 12 months after treatment.

Immediate conservative treatment included amoxicillin/clavulanic acid 2000/125 mg (Augmentin Plus, GSK, Madrid, Spain) every 12 h for 15 days, oral hygiene education and 0.12% chlorhexidine rinses every 12 h (Perio-Aid, Dentaid, Barcelona, Spain). The oncologist was consulted about discontinuation of bisphosphonates. At 15 days, partial closure of bone exposure without associated discomfort was observed; initial conservative treatment was maintained for another 15 days. At 30 days, soft tissue closure was complete, and the patient remained asymptomatic. Antibiotic treatment was discontinued, but 0.12% chlorhexidine rinses were maintained for an additional 15 days (Perio-Aid, Dentaid, Barcelona, Spain). At 12 months, there was normal soft tissue and no symptoms or signs of infection (Figure 2).

### 2.2. Case 2: Conservative Treatment Plus Surgery

An 80-year-old female non-smoker with type II diabetes, osteoarthritis and osteoporosis received oral ibandronate (Bonviva, Roche GSK, Madrid, Spain) for 12 months. She presented with discomfort in the left mandible 3 months after extraction of the second lower left molar.

**Figure 3:**
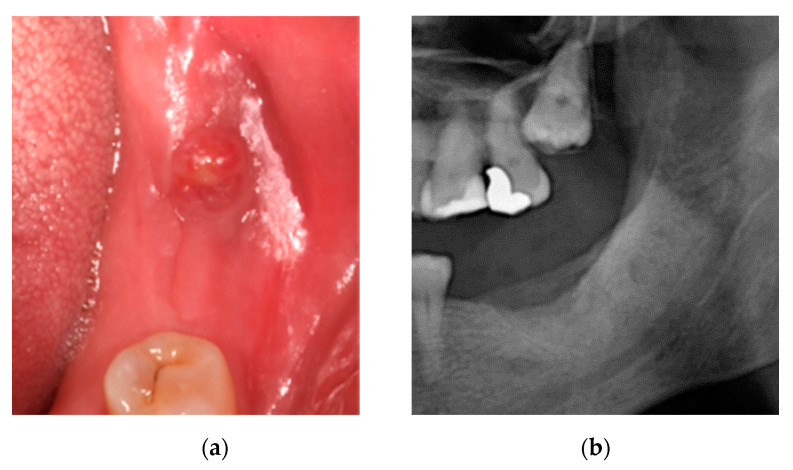
(**a**) Fistula in the jaw and (**b**) X-ray of the affected area.

Oral examination showed a fistula with inflammation and erythema of adjacent soft tissue without bone exposure and X-ray showed a lesion compatible jawmaxil osteonecrosis. The patient was diagnosed with MRONJ stage 2 (Figure 3). 

Immediate conservative treatment included amoxicillin/clavulanic acid (2000 mg + 125 mg every 12 h for 15 days) (Augmentin Plus, GSK, Madrid, Spain) and 0.12% chlorhexidine rinses (Perio-Aid, Dentaid, Barcelona, Spain) every 12 h for 30 days. The rheumatologist was consulted to discontinue bisphosphonates.

**Figure 4:**
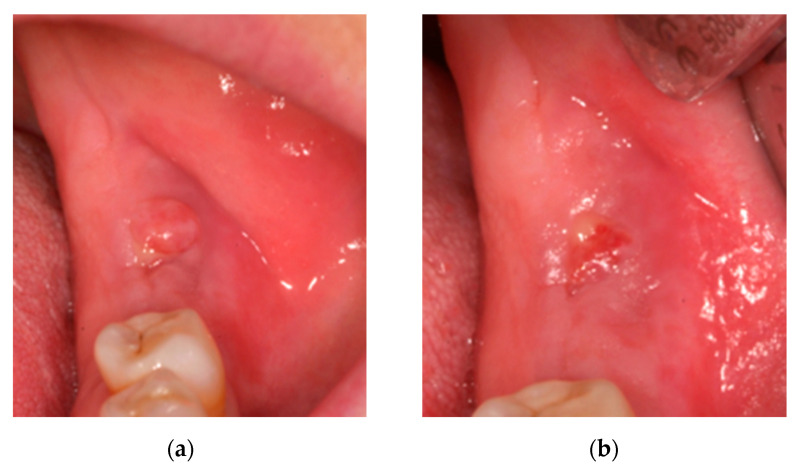
(**a**) Fistula at 15 days and (**b**) fistula at 1 month.

At 15 days, there was no discomfort and a significant reduction in fistula suppuration (Figure 4a), so treatment was maintained for another 15 days. At 30 days, there were no symptoms and an almost complete reduction in fistula suppuration (Figure 4b). 

**Figure 5:**
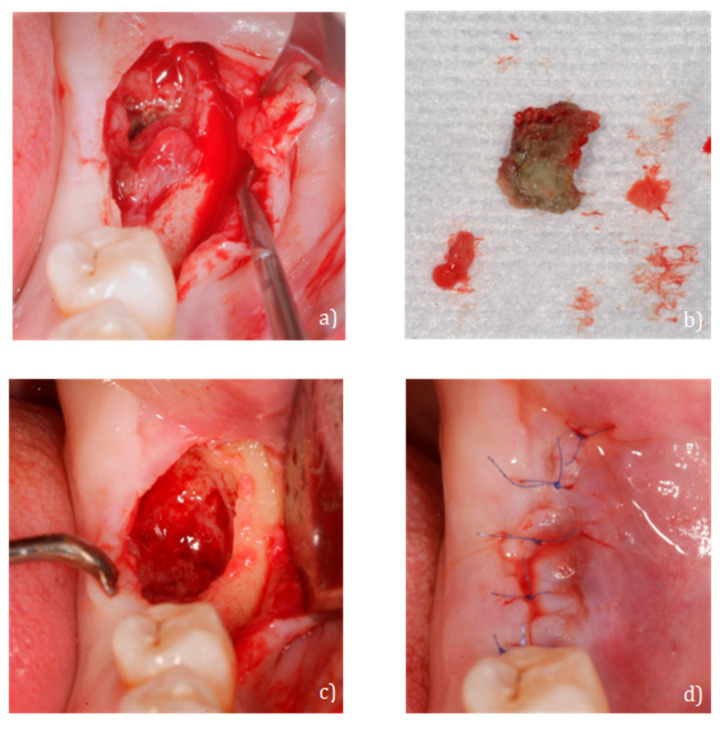
(**a**) Full thickness flap, (**b**) fragment extracted from necrotic bone, (**c**) surgical bed after curettage and irrigation and (**d**) monofilament suture 7/0.

Two months later, the patient reported discomfort and increased fistular suppuration, but without bone exposure. After re-establishing conservative treatment with amoxicillin/clavulanic acid (2000 mg + 125 mg every 12 h for 15 days) (Augmentin Plus, GSK, Madrid, Spain) and 0.12% chlorhexidine rinses (Perio-Aid, Dentaid, Barcelona, Spain) every 12 h for 30 days, minimal surgery for the removal of necrotic bone tissue was decided upon. After local anesthesia with articaine 4% + epinephrine (Inibsa, Barcelona, Spain), a flap was made at full thickness without discharges, necrotic bone tissue was removed, and smooth curettage of the bone walls with a dental spoon was made until bleeding was produced. The surgical bed was irrigated with serum and 0.12% chlorhexidine (Perio-Aid, Dentaid, Barcelona, Spain). The wound was stitched with 7/0 non-resorbable monofilament (Seralene, SERAG Wiessner Iberia, Madrid, Spain) (Figure 5).

**Figure 6:**
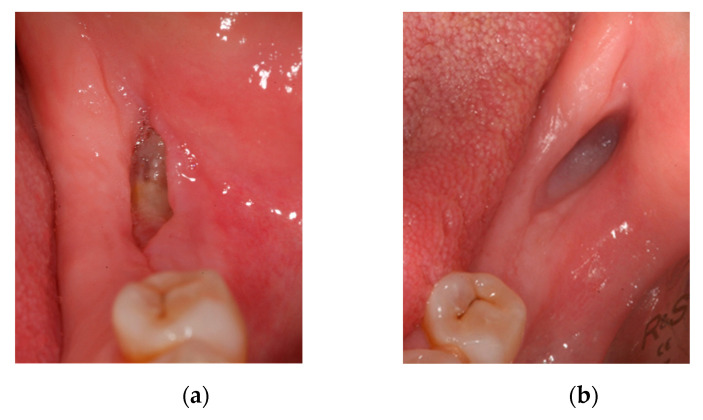

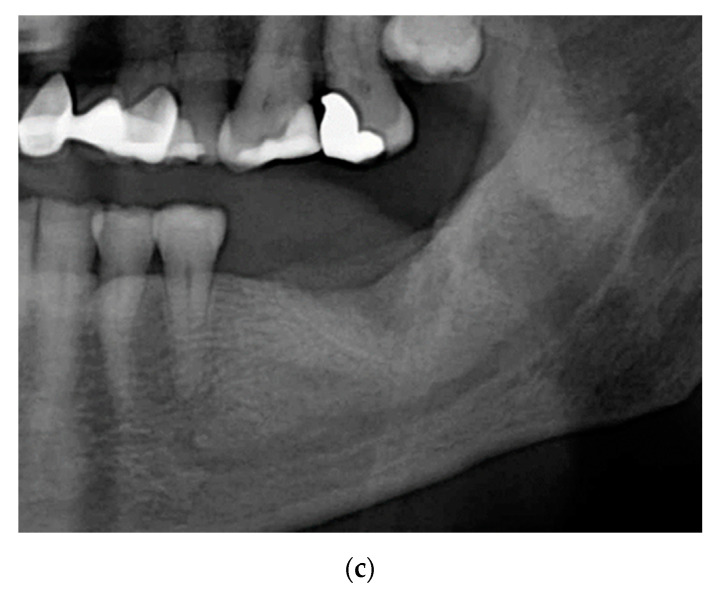
(**a**) Affected area 15 days post-surgery, (**b**) 30 days post-surgery and (**c**) X-ray 90 days post-surgery.

Fifteen days post-surgery there was no suppuration or discomfort, but the initial treatment was maintained for 7 more days (Figure 6a). At 30 days, there was closure of the soft tissue without signs of infection (Figure 6b) and 3 months later X-ray showed normal bone healing (Figure 6c). At 12 months, there was normal soft tissue with no symptoms or signs of infection.

### 2.3. Case 3: Conservative Treatment + SURGERY + PRF-L

**Figure 7:**
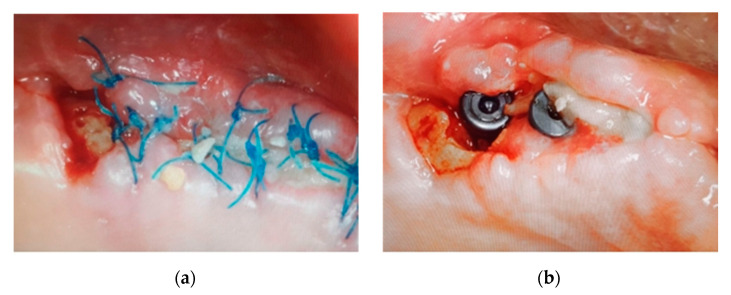
Bone exposure in the distal implant at 7 (**a**) and 15 (**b**) days post implant-surgery.

A 72-year-old female non-smoker with osteoarthritis and osteoporosis received denosumab 60 mg solution for injection (Prolia®, AmgenEurope B.V. Breda, Netherlands) every six months for two years. She developed discomfort and bone exposure after surgery for dental implants in the upper left jaw two weeks earlier (Figure 7). The patient was treated with amoxicillin 875 mg/clavulanic acid 125 mg every 8 h (Augmentin 875/125 mg, GSK, Madrid, Spain) and 0.12% chlorhexidine rinses every 12 h.

**Figure 8:**
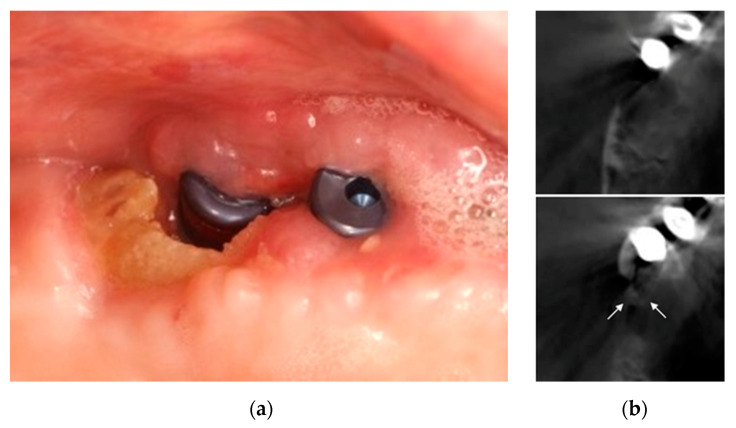
(**a**) Bone exposure at 30 days post implant-surgery. (**b**) White arrows: extension of bone necrosis at cross-sections at the crestal level and two millimeters towards apical on CBCT.

Oral examination showed a bone exposure of 8 × 4 mm^2^ without suppuration and associated with discomfort, with the surrounding soft tissue inflamed with exudate and bleeding; the implant was stable with no pain on percussion. The diagnosis was MRONJ stage 2. Conservative treatment was instituted including amoxicillin 875 mg/clavulanic acid 125 mg every 8 h (Augmentin 875/125 mg, GSK, Madrid, Spain) and 0.12% chlorhexidine rinses every 12 h (Perio-Aid, Dentaid, Barcelona, Spain) for 15 more days. The family physician was consulted to cease denosumab: the last 60 mg dose had been injected two months earlier. At 15 days, the patient reported no discomfort in the area; scans showed persisting bone exposure of the same size as at the beginning of conservative treatment, but with normal surrounding soft tissue, although with slight edema; the implant remained stable and painless on percussion. cone beam computed tomography (CBCT) was used to assess the extent of bone necrosis (Figure 8) and, as it affected only the exposed part, we resected the exposed necrotic bone without an access flap by with minimum curettage, screwing in of the healing plugs and continuing with antibiotics and chlorhexidine rinses for 15 days.

**Figure 9:**
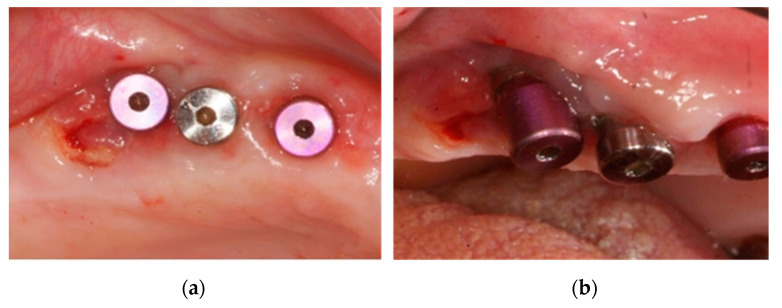
(**a**,**b**) Clinical situation at 45 days after removal of necrotic bone tissue and antibiotic therapy.

At two weeks, the patient reported discomfort again and although there was less bone exposure (Figure 9) exudate was observed. Surgery for the elimination of all necrotic bone, curettage and regeneration by platelet and leukocyte-rich fibrin (PRF-L) was carried out. 

**Figure 10:**
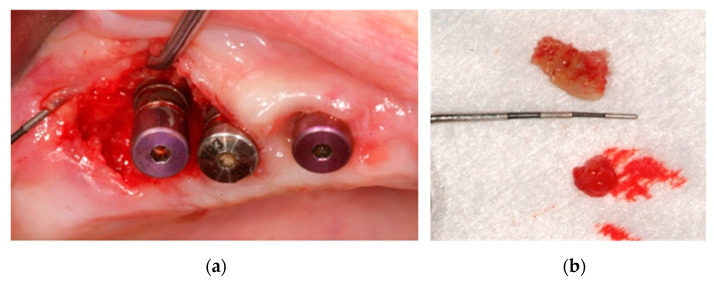
(**a**) Surgical access by a flap without discharge; (**b**) 7 × 4 mm bone fragment.

After local anesthesia with articaine 4% + epinephrine (Inibsa, Barcelona, Spain), a flap was made at full thickness without discharge, extending towards the distal. Necrotic bone tissue was eliminated, and smooth curettage of the bone walls was made with a dental spoon to generate bleeding from the bone walls of the defect. The surgical bed was irrigated with serum and 0.12% chlorhexidine (Perio-Aid, Dentaid, Barcelona, Spain) (Figure 10).

**Figure 11:**
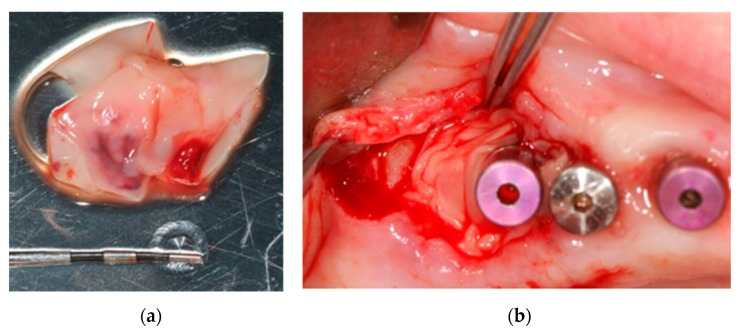
(**a**) PRF-L membranes; (**b**) Adaptation of PRF-L in bone defect and affected implant.

Before suturing the wound, several clots and PRF-L membranes obtained by centrifuge at 2700 rpm for 12 min of 40 mL of the patient’s blood were inserted (Figure 11).

**Figure 12:**
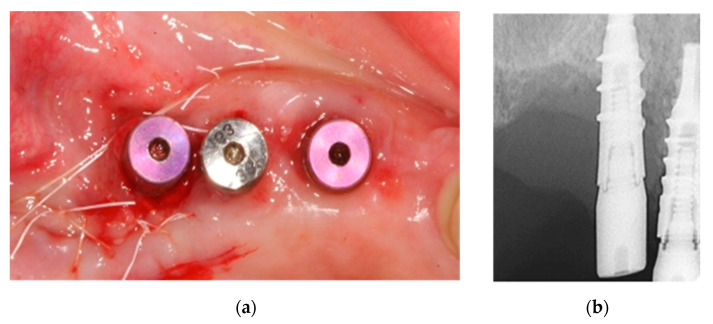
(**a**) Stress-free suture; (**b**) Resulting bone defect in post-surgical intraoral X-ray.

The wound was sutured with non-resorbable polytetrafluoroethylene 6/0 monofilament (Seramon®, SERAG Wiessner Iberia, Madrid, Spain) and an intraoral control X-ray taken (Figure 12). The same antibiotic therapy and 0.12% chlorhexidine rinses were maintained for a week.

**Figure 13:**
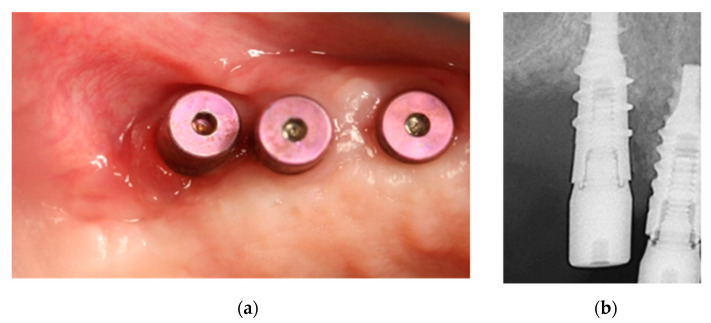
(**a**) Soft tissues with normal appearance at 12 months; (**b**) Radiographically stable bone tissue at 12 months.

After 7 days, sutures were removed, and an incidence-free healing and lack of symptoms were observed; 0.12 % chlorhexidine rinses were maintained for 15 days. Reviews at 1, 6 and 12 months showed normal healing of soft tissues and bone tissue (Figure 13). 

All patients gave informed written consent after a full description of the surgery in accordance with the guidelines of the Helsinki World Medical Association Declaration and the revision of the 2013 Guidelines for Good Clinical Practice.

## 3. Results

Twelve months after carrying out the protocols described by SECOM and AAOMS for the handling of stage 2 MRONJ, the three cases presented completely-resolved lesions. Clinical examinations at 1, 6 and 12 months showed a fully healed gum with a healthy appearance without pain or discomfort. Unlike case 1, case 2 did not initially improve with antibiotics and 0.12% chlorhexidine rinses but, after minimally-invasive resection of exposed necrotic bone there was complete remission of the lesion. Radiographically, slow but progressive healing of the affected bone area, with a normal bone structure, was observed. In case 3, in which the involvement was associated with the implant, which worsened the prognosis by communication between the oral medium and the necrotic bone tissue, a first attempt to remove the fragment was not successful and complete resolution was not achieved and a second surgical approach had to be used to remove more necrotic bone tissue and graft PRF-L, with antibiotics and 0.12% chlorhexidine rinses, resulting in normal tissue healing and favorable bone regeneration.

## 4. Discussion

The pathogenesis of MRONJ is not fully defined. Exaggerated osteoclastogenesis inhibition, and reduced bone replacement and angiogenesis may trigger osteonecrosis [7,17]. The process of bone turnover begins with the action of osteoclasts through bone resorption, followed by the formation of new bone tissue from osteoblasts. Bisphosphonates produce a significant increase in osteoclast apoptosis, while other antiresorptive drugs nullify their function and differentiation. Osteoclasts are activated, among other proteins, by RANK-L, the inhibitor of which is an antibody that prevents the binding of RANK-L with its nuclear receptor and therefore, preventing the function of osteoclasts. All of this results in a considerable decrease in bone resorption, regeneration and remodeling, increasing bone density and therefore reducing the effects of osteoporosis [8,25].

Although the optimal treatment of osteonecrosis is unclear, all scientific societies—AAOMS [7,8], SECOM [16], the Italian Society of Maxillofacial Surgery (SICMF) and the Italian Society of Pathology and Oral Medicine (SIPMO) [33], agree that minimally-invasive treatment, with the use of broad-spectrum oral antiseptics, is the best. Chlorhexidine gluconate, from the biguanide group, is the most widely used agent as it has a wide antimicrobial spectrum and substantivity, allowing chemical control of the biofilm. They are used in MRONJ stage 0, 1, 2 and 3 [34,35]. In the three clinical cases reported here, 0.12% chlorhexidine every 12 h, combined with antibiotics and painkillers, were important parts of treatment. In fact, in case 1, surgery was not necessary even though it was stage 2 MRONJ. In cases 2 and 3, surgery was necessary, together with chlorhexidine rinses and antibiotics, to achieve complete healing of soft tissues and bone, since no improvement was observed only with chlorhexidine. Bagan et al. [34] recommended, in addition to 0.12 chlorhexidine rinses twice daily, irrigation of the area affected every 72 h for 4 weeks and, if there was improvement, continuation of the rinses for one month.

The best antibiotic schedule for the treatment of osteonecrosis is not well defined. In fact, their use in both intermittent and continuous cycles to prevent osteomyelitis and superinfection of the soft parts has been proposed [36,37]. Some authors have recommended clindamycin due to its efficacy against Gram-positive flora and its affinity for brain tissues [38], although Marx et al. [36] discouraged it as single therapy, and recommended penicillin derivatives as first choice drugs. The antibiotic administered should cover Actinomyces, Fusobacterium, Eikenella, Bacillus, Staphylococcus, Streptococcus and Treponemas [39]. Of all these, the bacterium that stands out is Actinomyces, as it has been observed in histological sections of osteonecrosis, although it is unknown whether it plays a primary (co-producer of MRONJ) or secondary role (colonizes the necrotic bone once in place), due to the difficulty of microbiological isolation, since no biopsy is usually performed in patients receiving bisphosphonates, for obvious reasons [40].

Ideally, an antibiogram would be performed but, if not possible, the most appropriate antibiotics in the acute infection phases are beta lactams (amoxicillin or amoxicillin + clavulanic acid) [41]. According to SECOM [16], the guideline should be amoxicillin 875 mg/125 mg clavulanic acid or amoxicillin 2000 mg/125 mg and, in case of allergy to penicillin, clindamycin 300 mg every 8 h for 7 days, or levofloxacin 500 mg every 24 h, which have shown good results [37]. Other reports used clindamycin for two weeks, followed by amoxicillin-clavulanic acid for two weeks, and then penicillin G depending on the culture, although without specifying for how long [41]. In cases of beta lactam allergy, macrolides (azithromycin) or quinolone (ciprofloxacin or levofloxacin) are also used alone or in combination. If these are not effective, clindamycin or metronidazole [8,42] is added. In our cases, good results were obtained following the SECOM protocol, and administering 2000 mg/125 mg of amoxicillin- clavulanic acid in cases 1 and 2, and amoxicillin-clavulanic acid 875 mg/125 mg in case 3 for 15 days, and prolonging treatment for 15 days if no improvement was observed.

The non-surgical treatment in stage 3 patients, consisting of systemic antibiotics, pentoxifylline 400 mg (which promotes blood circulation), tocopherol (vitamin E) IU twice daily and 0.12% chlorhexidine rinses four times daily, significantly improves pain, symptomatology and bone exposure [43].

If mycosis associated with osteonecrosis is suspected, nystatin or fluconazole should be used. Some studies also prescribed penicillin G + IV metronidazole [44], levofloxacin + metronidazole [45], piperacillin + tazobactam or imipenem + cilastatin [46,47], with favorable results. Mycosis was not suspected in any of our three cases, so no antifungal treatment was necessary.

Reports suggest antibiotics should be given for one week [48], ten days [49], fifteen days [50], three or four weeks, or until healing is complete [47]. Most studies agree that antibiotic treatment should be long-term until clinical remission of osteonecrosis-related signs and symptoms is achieved [47].

Antibiotics fail in some cases, possibly due to the difficulty of reaching a target in an environment with little vascularization. In these cases, if symptoms are mild, some authors suggest bone debridement and closing with mucous flaps, always with antibiotic treatment [51]; this involves the removal of the exposed necrotic bone by surgical curettes, and it may be difficult to obtain margins with viable healthy bone: bisphosphonates or denosumab have little influence [45,52]. Antibiotic-impregnated membranes that may act as local anti-infectious drug release systems, which not only fill non-vital space after surgical debridement, but also provide high concentrations of antibiotics at the potential infection site, without increasing serum antibiotic levels, are currently being studied [53]. For example, using a collagen matrix impregnated with gentamicin as a topical supplement for perioperative antibiotic prophylaxis. The gentamicin-collagen sponge used by Chia et al. consists of a type I purified bovine collagen matrix impregnated with gentamicin sulfate 2.0 mg/cm, and the authors suggest it reduces the risk of postoperative infection after being applied in 92 patients [54].

When surgery is used, a signal that may indicate the “healthy” bone margin is normal bleeding in the bone, which indicates there is sufficient potential for healing, although this is not always the case, and in many cases it cannot serve as a golden rule [55]. In addition, the use of ultraviolet light after prescribing tetracycline (250 mg 4 times daily for 3 days) or doxycycline (100 mg 2 times daily for 10 days) has been suggested as a means of detecting the extent of osteonecrosis and delimiting the margin of the resection successfully, even for surgical debridement [56]. Most common minimally invasive surgery technique is removal of symptomatic bone sequestration with minimal injury to the soft tissues [41]. However, the removal of necrotic bone may, in some situations, worsen the clinical picture. The use of surgical ultrasound in bone removal has shown good results as it allows careful cavitation due to the vibration around osteonecrosis, causing degradation of bacterial complexes and favoring revascularization and wound healing, although more experimental studies are needed [57,58].

In addition to performing careful surgery technique, recent studies have also used growth factors such as platelet-rich plasma (PRP) or platelet and leukocyte-rich fibrin (PRF-L) in order to stimulate angiogenesis and the repair of local bone tissue [59]. In this procedure, all affected alveolar bone is removed, leaving only the basal component, grafting the PRP or PRF-L, and attempting primary closure of the mucosa [60,61]. Dinca et al. (2014) used PRF-L in patients with jaw osteonecrosis stage 2 following intravenous bisphosphonate therapy in the alveoli post-extraction [62]. The sample used was small and the study had limitations, but there were no postoperative complications in any of the 10 cases studied and after 30 days there was no evidence of bone exposure [62]. In our study, PRF-L membranes were applied in the second surgical approach in patient 3, achieving better results than in the first approach where PRF-L was not applied, and resulting in complete soft tissue healing and bone regeneration. The use of platelet- and leukocyte-rich fibrin membranes in patients with MRONJ appears to be a promising treatment due to the association between MRONJ and bone remodeling suppression, antiangiogenic effects, reduced immune response and soft tissue toxicity; however, further research is needed to confirm these effects [63].

Another therapeutic option for the treatment of MRONJ is the use of stem cells. In an experimental study in rabbits under treatment with bisphosphonates and immunosuppressants, stem cells prevented osteonecrosis and significantly improved healing after tooth extraction and local transplant of stem cells derived from adipose tissue [64]. There are few human studies of this therapy: there is a report of a 71-year-old patient with multiple myeloma in the jaw, who was treated with autologous bone marrow stem cells, platelet-rich plasma, beta tricalcium phosphate and demineralized bone matrix. At 6 months, healing and bone formation had improved. The authors concluded that cell therapy could be applied in patients with refractory osteonecrosis [65].

This case series suggests the effectiveness of the various protocols published for the treatment of stage 2 MRONJ, although the small number of patients included is a limitation. Therefore, randomized clinical trials with a control group and more patients included, using the same protocol and treatment parameters, are recommended.

## 5. Conclusions

Conservative treatment with antibiotic therapy, chlorhexidine rinses, minimally invasive surgery with necrotic bone resection and the application of PRF-L membranes has been shown to be an effective treatment for stage 2 MRONJ.

Studies of denosumab-related jaw osteonecrosis have shown it can be resolved more quickly with the suspension of the drug for 6 months compared with bisphosphonates, but during treatment, denosumab resulted in a higher incidence of MRONJ with less trauma, and sometimes spontaneously; further studies are needed.

The novel therapeutic options for the treatment of MRONJ, such as platelet and leukocyte-rich fibrin (PRF-L) represent improvements in soft tissue healing and bone regeneration, although more research is needed to confirm their effectiveness.

## Figures and Tables

**Table 1:** Grades or stages of MRONJ according to the AAOMS [8].

MRONJ Grades	Description
At risk:	No apparent necrotic bone in patients treated with oral or intravenous anti-resorptive or anti-angiogenic agents.
Grade 0	Without necrotic bone exposure, but with clinical findings, radiographic changes, and nonspecific symptoms.
Grade 1	Exposure of necrotic bone and fistulas, asymptomatic and without signs of infection.
Grade 2	Exposure of necrotic bone and fistulas associated with signs of infection; pain and erythema in the affected area with or without purulent drainage.
Grade 3.	Exposure of necrotic bone and fistulas associated with pain, infection and ≥1 of the following: exposed necrotic bone extending beyond the alveolar bone (i.e., lower border and branch of the jaw, maxillary sinus, and maxillary zygoma) resulting in pathological fracture, extraoral fistula, oroantral or oronasal communication, or osteolysis extending to the lower border of the jaw or the floor of the sinus [8]

**Table 2:** Recommended treatments according to the grade or stage of MRONJ.

MRONJ Grades	Treatment
At risk:	- No treatment indicated. - Preventive patient education.
Grade 0	- Systemic management, including painkillers and antibiotics.
Grade 1	- Clinical follow-up with quarterly antibacterial mouthwashes. - Patient education and review of indications for continuous therapy with bisphosphonates.
Grade 2	- Symptomatic treatment with antibiotics. - Antibacterial mouthwash. - Pain management. - Debridement to relieve soft tissue irritation and infection control.
Grade 3.	- Antibacterial mouthwash. - Antibiotic therapy and pain management. - Surgical debridement or resection to alleviate long-term infection and pain.

**Table 3:** Treatment protocol recommended by SECOM for MRONJ.

MRONJ Stages	Treatment
Stage 1	Quantification in millimeters of the exposed area. If possible, stop treatment with anti-resorptive or antiangiogenic drugs. Rinses with 0.12% or 0.2% chlorhexidine every 12 h for 15 days. Control at 15 days: - Equal or smaller size: maintain treatment for another 15 days. - Increased exposure: apply stage 2 treatment. Control at one month.
Stage 2	Points 1–3 as in stage 1. Empirical antibiotic therapy (when no culture or antibiogram is available): - Amoxicillin/clavulanic acid 2000/125 mg, every 12 h for 15 days. - Allergic patients: Levofloxacin 500 mg every 24 h for 15 days. - Alternative: Azithromycin. Administer oral nonsteroidal anti-inflammatory drugs (NSAIDs). Control at 15 days: - Improvement: move to stage 1 treatment. - Equal or worse: maintain treatment for another 15 days and request computed tomography (CT). Control at one month: - Improvement: move to stage 1 treatment. - Equal or worse: move to stage 3 treatment.
Stage 3	Point 1–3 as in stage 1. Point 2 and 3 as in stage 2. Under local anesthesia, eliminate bone sequestration and extraction of teeth involved if necessary, irrigation of the surgical bed with 0.12% chlorhexidine and suture with resorbable material. Control at 15 days: - Improvement: maintain antibiotics, anti-inflammatories and rinses for another 15 days. - Equal or worse: maintain antibiotics, anti-inflammatories, and rinses for another 15 days. - Control at one month - Improvement: implement preventive measures and reinstate treatment with anti-resorptive or antiangiogenic drugs. - Equal or worse: new conservative surgery in serious circumstances: Pathological fracture: curettage of necrotic bone tissue and reconstruction plate (avoid grafts). Involvement of inferior border: block resection and reconstruction plate. Extraoral fistula: debridement to eliminate necrotic bone causing mucous irritation.
